# A Manual of Comparative Dental Anatomy for Dental Students

**Published:** 1899-07

**Authors:** 


### THE NATIONAL ASSOCIATION OF DENTAL FACULTIES.

THE annual meeting of the National Association of Dental Faculties will be held at Niagara Falls, beginning July 28, at 10 A.M., 1899, and will continue until the 31st.

The Executive Committee of this body will meet on Thursday, the 27th, at ten o'clock, for the preparation of business.

This meeting will be an important one, and it is essential that all schools should be represented.

---

### Bibliography.

A MANUAL OF COMPARATIVE DENTAL ANATOMY FOR DENTAL STUDENTS. Prepared by request of the National Association of Dental Faculties, and adopted as a text-book for colleges August 27, 1898. By Alton Howard Thompson, D.D.S., Topeka, Kansas, Professor of Dental Anatomy, Human and Comparative, in the Kansas City Dental College, Kansas City, Mo. The S. S. White Dental Manufacturing Co., 1899.

The best evidence that can be given of the advance in the dental curriculum is that presented by this text-book, prepared by request of the Association of Faculties. When, in the past, this subject of comparative anatomy was made part of the study in the dental schools, it was received, both by students and faculties, with indifference, and the teacher felt forced to reduce the number of his lectures or abandon the subject altogether. Several, including the reviewer, labored for years to create an interest in this important subject, but the success, until recent years, was not commensurate with the efforts made. That there has been a marked change in this feeling is very gratifying, and now dental colleges are seeking men competent to teach this branch. The author recognizes this change, and in his preface says, "The time was when it was necessary to apologize for the intrusion of comparative dental anatomy into the curriculum of dental education, but it is a matter of congratulation that the value of this branch, as an element in our professional education, is now generally recognized. . . . It is also recognized that this study furnishes the only scientific elucidation of the origin and principles of these forms and functions which had heretofore been taught by the study of the human teeth alone."

The book has been kept within reasonable limits, 174 pages, the author having, apparently, in mind that a large book, with padded text, is a serious evil. Clearness and brevity is not recognized as it should be by dental authors.

The book is divided into nine chapters: Chapter I. on "General Zoology and Comparative Anatomy," and then follow "Teeth in General, of Invertebrates, Vertebrates, Fishes, Reptiles, Mammals, Higher Apes, and Man." This covers a wide field, but the subject-matter is so lucidly stated that there is left but little to be desired. While comparative anatomy cannot and ought not to be taught from books alone, much can be gained from a work of this kind to direct the course of study.

The care given to the compilation of this book disarms criticism. In all the chapters there is an evident care to follow accepted lines of observation. Originality is not to be looked for, nor would it be desirable in a book strictly intended for students.

It seems to the reviewer that the author assumes a too positive statement in discussing the development of forms on page 47. He states that "the premolar and molar teeth were evidently developed by the duplication of cones by fission or addition. . . . Thus the cones were duplicated and tubercles added to form multitubercular types." This corresponds with accepted ideas and may have been the true evolutionary process, but that it has been proved is not so certain.

In the effort to combine brevity with clearness of description, the author, at times, fails to enter sufficiently into detail. This is especially noticeable in the description of the sea-urchin, "Aristotle's lantern." This complicated dental apparatus deserves more than a paragraph and the limited description of the number of the teeth.

In giving the names and functions of teeth, on page 50, he states that "the premolars are the half molars just distally of the canines. These are called the bicuspids in man." While it is true that the common and universal name is bicuspid, it is a question whether this term should not give place to premolar, or else call the molars by the number of cusps. As this is impractical, it would seem better to drop this common term in teaching.

The series of questions following each chapter do not, in the opinion of the reviewer, add to the value of the book. Students should not be taught to study comparative anatomy by memorizing each chapter. Anatomy cannot be studied that way, but the temptation is always present, where these questions exist, to take that course, as a short cut to knowledge.

This book will not, probably, have any competition in this country, and it is to be hoped that, with some minor changes, it will remain not only as a text-book, but as an incentive to dental faculties to broaden this study, the only sure foundation for an exact knowledge of the teeth of man.

